# The traps of calling the public health response to COVID-19 “an *unexpected war* against an *invisible enemy*”

**DOI:** 10.1057/s41271-020-00237-y

**Published:** 2020-07-20

**Authors:** Elena N. Naumova

**Affiliations:** Friedman School of Nutrition Science and Policy, Tufts University, 150 Harrison Avenue, Boston, MA 02111 USA

Any topic related to COVID-19 is too vast for a single editorial. This pandemic has revealed the bare-bones deficiencies of national health systems. The crisis has unearthed matters many might have thought the public health community had already resolved. The delayed local and regional emergency responses, the supply chain crash, the inequality in access to protective gear, the discordant and mixed messages of health officials and political leaders—in short, all aspects of the COVID-19 pandemic deserve serious attention and will be subjects for discussion and soul-searching for many years to come.

Yet, one recurrent topic keeps puzzling me: the rhetoric for responding to pandemics is equated with an unexpected war against an invisible enemy. Below I take up each element of this rhetoric separately: the *invisible enemy*, and the *unexpected war* to dissect their worth. I argue that this entire concept brings to public health and public health policy more harm than help. I call for keeping the role of public health professional clear: to protect public health using proven scientific evidence and best practices and to serve to community at large.

By instilling that a cause of infection is invisible, we are implicitly rejecting the science. By presenting a virus as an enemy we impede responsibility humans bear for driving patterns of disease. We refuse to accept that the virus—a cause of infection—is a part of the environment, which we, humans, are altering to suit our needs. By insisting that the pandemic was not expected, we deny the warning signs and the facts that limited preparedness efforts had transformed initial outbreaks into a massive pandemic. Most importantly, by focusing on the ‘war on a virus,’ we deemphasize the role of evolution—and our role in shaping the evolution-inspired tolerance to infection.

The language we use to describe the response to pandemic does matter. In our efforts to rally and retaliate against the common cause, we are losing sight of the infrastructural collapse, the failure of early warning and detection, and reactionary behavior toward mitigating infectious disease outbreaks. The war-inspired rhetoric distracts public attention from the issues of critical importance at a very high price due to denial and ignorance.

## An *invisible enemy*? No, this virus is real, visible, and part of environment we had created

The theory of miasmas popular in the 1800s accepted the notion that a poisonous vapor or mist made up of *particles* from decomposing material could cause disease. In the early twentieth century the miasma theory was replaced by the germ theory, further verifying the cause of infection—not particles, but germs. In the last two hundred years, scientists provided ample evidence for real disease-causing agents—bacteria, protozoa, viruses—and the role of humans in unleashing multiple routes of transmission.

Scientists and public health workers made tremendous strides to produce vaccines, treatments, and preventive measures to create the barriers against spreading infections. Yet, the progress depends on continuous targeted efforts in reducing factors that lead to the wide spread of infections. Notably, the most devastating infectious outbreaks are typically amplified by the man-made conditions, like poor hygiene and sanitation, crowding, malnutrition, civic unrest, and income inequality.

From the start of the COVID-19 pandemic, news, media, and specialized literature circulated images of a coronavirus—an agent causing corona virus infectious disease (COVID-19). Reputable sources, such as the US Centers for Disease Control and Prevention (CDC), had posted highly magnified, digitally colored transmission electron microscopic images that revealed ultrastructural details exhibited by a single, spherical shaped, Middle East respiratory syndrome coronavirus (MERS-CoV) virion [1]. No mysteries, no *invisible*, just need of a powerful microscope and experts able to create an illustrated visualization aiming to inform and educate the public about the disease. Why then the insistence on the ‘invisible’ even figuratively? And why especially when these images are produced by a reputable organization, such as the United States National Institute of Allergy and Infectious Diseases (NIAID)?

This colorful 3D rendering of a spiky fuzzball has spread around the world as fast as the infection. The ‘mug’ shots isolated a culprit, one that has been targeting human health, and demanding global response. The virus became an enemy and joins the list of ongoing wars on obesity, guns, cancers. I wonder if along with calling for attention to important public health matters we are also shifting the focus to demonizing pathogens, narrowing of our understanding of disease transmission, and diluting human responsibility for acquiring, carrying, and distributing disease among ourselves. By blaming the virus, humans appear to be less culpable for failures in early warning, detection, planning, programming, interventions, and for creating conditions for a virus to thrive. In the twenty-first century, even a metaphoric use of an *invisible enemy*, recklessly amplified by those with the access to large audiences, denies the knowledge and progress achieved over 200 years of science.

Many common infections, such as influenza (flu), salmonellosis, and rotaviral infections, manifest themselves by seasonal outbreaks. Flu is notorious for reminding us with remarkable consistency that humans are part of a complex evolutionary process. In this process, resilient species are not those better at *fighting against* others but rather those who are better at *living with* others. The concept of tolerating an infection, an evolutionary-ingrained strategy, is not new. Steady worldwide growth in life expectancy and quality of life has been achieved by reducing exposure to pathogens, improving nutrition, and implementing vaccination programs. Seasonal infections are the consistent reminders of ongoing evolution. The human genetic makeup reflects a process in which selection of specific Human Leukocyte Antigen (HLA) types amounts to a footprint of thousands of years of building evolutionary-based tolerance—that protects humans. We are who we are because of the centuries of evolution and several decades of active boosting of immune responses with vaccines. Well-designed vaccination strategies offer well-controlled boosts for humans’ immune systems-and these drive future health responses to seasonal outbreaks. By understanding the seasonal disease patterns, we learn how to make vaccination strategies efficient: when and whom to vaccinate and how to measure the success and failures. By anticipating seasonal reoccurrence of COVID, we should find ways to protect the public and provide services our communities deserve.

Communities and their governing systems, which we, humans, have built, can amplify and dampen the outbursts of infection. We, humans, are creating conditions in which the distribution of risks among those exposed is unequal. We, humans, are making the decisions about how to distribute protective gear, mobilize fiscal resources, or train medical personnel [2]. When the decision chain works correctly, we all receive the right protection according to the dose, intensity, and duration of potential exposure. Yet, when the decision chain is broken or not even established, a sudden search for an invisible enemy starts, and war rhetoric easily pushes forward an invisible culprit. The slogan of ‘war on a virus,’ is easy to turn into a war on scientists, reporters, whistleblowers [3]. The rhetoric of war is divisive and distracts from challenges of finding solutions and building consensus.

## An *unexpected* pandemic? No, scientists predicted the crisis and the public health community demanded preventive actions

As with many public health research outlets, the *Journal of Public Health Policy* has published several warnings. In 2018, Fedson wrote: “When the next pandemic virus emerges, the world might be confronted with a social, political, and economic crisis of unimaginable dimensions.” [4] He based these warnings on thoughtful examination of viral and host evolution, conditions that create challenges for survival of both the virus and the host, and conditions that help the host to tolerate the virus [5]. The host–pathogen relationships are grounded in iterative evolutionary processes. The war rhetoric distracts public from pragmatic tasks aiming at preventing pandemics through strengthening the global health security agenda, restricting unsanitary wildlife markets, and decreasing environmental degradation.

The issue of inadequate personal protective equipment that compromised the ethical responsibility of healthcare workers to protect their patients is no longer only a feature of low- and middle-income countries. Access to health care services and treatment, protective equipment, and affordable testing has been plagued by preexisting inequalities worldwide. While public health professionals strive to adapt to new norms of high urbanization and globalization of economic ties and trade, the public health field depends on governmental willingness to learn from lessons of past epidemics. So far, the pandemic has revealed when and where decision makers opt for denial—and use war rhetoric rather than responsibility for failed preparedness plans and proactive policies.

## The language we use to describe the response to pandemic does matter

War analogies are often called upon in responding to important socio-emotional needs, like a call for public service, a sacrifice for a common good, an appeal to compassion, empathy, and rightful support of those who are on the frontlines. These socio-emotional responses help us to cope, mobilize resources, change the course of action in reaction to natural disasters and large-scale man-made accidents.

During the pandemic, medical personnel, nurses, public health practitioners, and first responders are considered to be “on the front line” or “battling diseases head on.” By framing the pandemic in military terms, officials are communicating the gravity of this public health crisis, calling for personal sacrifice, and justifying interventions required to control the spread infection. Yet, this flawed parallel also increases fear, anxiety, and panic and is likely to have lasting consequences. When the war references are applied to pandemics, the appeal to human emotions can be easily abused by shifting public attentions from thoughtful and comprehensive strategies of balancing risks and the needs for keeping communities functioning to impulsive reactions. Over decades this journal and many others have shown examples of effective public health communication strategies for bringing public attention to important issues, mobilizing resources, and advocating for public health policy reform. This pandemic is a reminder that the solutions we are seeking for one problem should not create future problems.

I believe this entirely war-driven rhetoric brings more harm than good to public health and public health policies. The harm stems from the targeted and intentional distraction of public attention from the issues of critical importance by searching instead for a scapegoat. Attacking scientists who need time and resources to find solutions is less risky than accepting responsibility for not providing needed time and resources. Pointing at the enemy is easier than revealing and understanding the complexity, inequalities, interdependence, and ethical challenges of any decision we make, especially in rapidly changing situations full of uncertainties.

The agenda for public health research and practice remains: to investigate, build, implement, and maintain strategies for improving overall population health and resilience, and thus, to gather and use, not hide or destroy, the information that can drive improvement. It implies emphasis on prevention, protection, and control by boosting immune systems at the individual level, by boosting population resilience at the community level, and by boosting coordination, cooperation, and collaboration on the national and international levels. There is no room for war and there should be no incentive to support misleading rhetoric. As we are seeing, the cost in human lives is way too high.

Elena N. Naumova, Editor-in-Chief.
